# Advances in Achilles Tendon Tissue Engineering: Integrating Cells, Scaffolds, and Mechanical Loading for Functional Regeneration

**DOI:** 10.3390/bioengineering12121346

**Published:** 2025-12-10

**Authors:** Sedeek Mosaid, Paul Lee, Yousif Jihad

**Affiliations:** United Lincolnshire Hospitals NHS Trust, Lincoln LN2 5QY, UK; leep@mskdoctors.com (P.L.);

**Keywords:** Achilles tendon, tissue engineering, scaffold, mesenchymal stem cells, growth factors, mechanical loading, 3D bioprinting, regenerative medicine

## Abstract

Achilles tendon injuries are among the most frequent and debilitating musculoskeletal conditions, often resulting in incomplete healing and functional deficits. Conventional repair techniques primarily restore structural continuity but rarely achieve full biomechanical or histological regeneration. Recent advances in tissue engineering have introduced innovative strategies combining biomimetic scaffolds, cellular therapy, growth factors, and mechanical loading to promote regenerative rather than fibrotic repair. This review summarises the current understanding of Achilles tendon biology and healing mechanisms, with a focus on the integration of stem cell technologies, scaffold design, and mechanobiological conditioning. Various scaffold systems, including natural, synthetic, hybrid, and hydrogel-based constructs, are evaluated for their biocompatibility, mechanical performance, and tenoinductive potential. Preclinical studies demonstrate that mesenchymal stem cell (MSC)-loaded scaffolds exhibit significantly enhanced biomechanical outcomes in tendon defect models, including improved tensile strength, organized collagen I deposition and aligned fibre architecture in repaired constructs. While preclinical results are promising, clinical translation remains limited by regulatory, economic, and methodological challenges. Future research should prioritise standardised protocols, long-term functional outcomes, and interdisciplinary collaboration.

## 1. Introduction

Tendons are fibrous connective tissues that attach muscles to bones and transmit forces. They store energy, maintain posture, and produce joint movement. These functions require tendons to have high tensile strength and withstand large compressive forces [1,2,3].

The Achilles tendon (AT), also known as the calcaneal tendon, is the largest and strongest tendon in the body. It is formed by the merging of the gastrocnemius and soleus muscle tendons. During running, the AT is subjected to large forces and experiences a strain reaching 12.5 times the body weight. The AT is the most injured tendon, accounting for approximately 40% of all ruptures [4,5].

Tissue engineering is defined as “the application of the principles of biology and engineering and appropriate use of chemicals, cells, and factors to develop substitutes that restore, maintain, or improve tissue function” [6]. The regeneration of a tendon with the same composition as the original uninjured tendon may be critical for proper management of tendon injuries [7].

Tissue engineering approaches have revolutionised AT injury management by promoting the regenerative healing of the native tendon structure and function using biomimetic scaffolds, stem cells, growth factors, and mechanical stimulation [8,9].

Conventional repair methods usually lead to the formation of fibrotic scar tissue with poorer mechanical properties than normal tendon tissue. Conversely, tissue engineering approaches aim to guide a regenerative healing process that restores the aligned collagen structure and biomechanical properties of healthy tendon tissue. This is especially critical for the AT, which has a poor blood supply and an inherently low healing potential [8,9].

## 2. Macroscopic Structure

Establishing baseline compositional characteristics for normal tendons is essential to set suitable standards for healing and identify effective strategies for successful functional tissue engineering [10].

The AT is the most robust tendon in the human body, with a vital role in human upright walking. It links the triceps surae muscle, which includes the medial and lateral heads of the gastrocnemius muscle and the soleus muscle, to the calcaneus bone tuberosity, forming the back shape of the calf [11].

In 65% of individuals, the plantaris muscle tendon is laterally associated with the AT. The AT facilitates the transfer of forces generated by the triceps surae muscle, which are sufficiently substantial to manage various body weight loads, acting as the primary agent at the ankle joint to mediate plantar flexion. Consequently, the tendon is heavily stressed in sports involving jumping and rapid accelerations and stops, such as football, basketball, and running [11].

The AT is approximately 15 cm long. It has a characteristic spiral internal configuration in which fibres from the medial head of the gastrocnemius rotate laterally across the AT, and fibres from the lateral head rotate medially. Fibres from the soleus muscle remain centrally placed. The AT’s spiral configuration enables it to store energy and elastic recoil during running and jumping. Like most tendons, the AT is relatively hypovascular [12,13].

The blood supply to the AT is primarily from the posterior tibial artery, with a contribution from the fibular (peroneal) artery. These vessels provide perforating branches that course through the paratenon. Three vascular regions have been defined based on these supply branches, with the proximal third and distal insertion areas being primarily supplied by branches of the posterior tibial artery and the mid-portion, 2–6 cm above the calcaneal insertion, being supplied by the fibular artery [14,15]. The mid-portion has been demonstrated to be relatively hypovascular and is more susceptible to degeneration and rupture. Additionally, the torsional arrangement of the subtendons in the mid-portion has been linked to greater compressive loading in the central hypovascular core. It is proposed that these morphological factors are involved in the high prevalence of tendinopathy and the lack of a healing response at this site [11,14,16].

## 3. Microscopic Structure

The AT is a hypocellular tissue and mainly comprises the extracellular matrix (ECM). Collagen type I accounts for approximately 70% of the dry weight of the AT and is the main contributor to the tensile properties of the tendon [11,17]. The ECM also contains elastic fibres, a small amount of proteoglycans (1% of dry weight), and glycoproteins, including tenomodulin and tenascin-C, which are implicated in the regulation of cellular behaviour and tendon healing [11]. The composition of the ECM differs between the mid-portion of the tendon and the enthesis; the former is fibrous, while the latter is fibrocartilaginous with distinct biomechanical properties. Collagen types I, III, V, and VI, as well as small leucine-rich proteoglycans such as decorin, biglycan, fibromodulin, and lumican, are found in the mid-portion of the tendon and the enthesis but not in equal proportions. Type II collagen and aggrecan are present only in the enthesis, and versican is present only in the mid-portion of the tendon, a distribution thought to be related to their functions in resisting tension and compression, respectively. Consequently, the highly aligned type I collagen fibres are the primary contributors to the tendon’s mechanical properties. Conversely, type III collagen forms a finer fibrillar network to support the former. The proportion of type III collagen is inversely correlated with tensile strength, with an increase in type III collagen leading to a decrease in tensile strength [11,18].

Tenocytes are spindle-shaped fibroblast-like cells scattered through the collagenous matrix of the AT and located in fascicular and interfascicular compartments. They are oriented parallel to collagen fibres and are the principal cell type maintaining the ECM homeostasis. Tenocytes are also mechanosensitive, responding to mechanical stimulation via integrin-mediated signalling, gap junctions, and stretch-activated ion channels, which activate MAPK/ERK, TGF-β, and Wnt/β-catenin pathways that control matrix turnover/remodelling and proliferation [11,19,20].

The ECM contains several essential non-collagenous components, including elastin, decorin, biglycan, lubricin, and water. These components are responsible for the tendon’s viscoelastic properties, including stress relaxation, creep resistance, fibril sliding, and tissue recoil. Elastin, a key ECM component less abundant than collagen, is highly important in energy-storing tendons. Elastin returns rapidly to its original length after deformation and is essential to repetitive activities such as running and jumping. Decorin and aggrecan are proteoglycans that maintain collagen fibril spacing, influence tissue hydration, and control interfibrillar shear stress. These functions provide resistance against mechanical fatigue [11,21,22].

The crimp pattern of collagen fibrils is an ultrastructural feature of tendons, enabling them to function as mechanical shock absorbers. The ability of tendons to straighten when a load is applied enables energy dissipation. The straightening of crimps as a load is applied enables the tendon to transmit force efficiently with limited structural compromise. The wavy morphology of tendon crimps is important for efficient force transmission and a vital part of the tendon’s mechanical response. Changes in crimp morphology (such as reduction in crimp amplitude or wavelength) can result from ageing or pathology. This can result in compromised mechanical performance and increased susceptibility to injury [11,23,24].

From a translational bioengineering perspective, the precise characterisation of the cell–matrix organisation, mechanotransduction pathways, and hierarchical viscoelastic network of the AT is critical for developing targeted regenerative therapies. Engineering approaches should focus on recapitulating these features using biomimetic scaffolds, cell-seeded constructs, and mechanically preconditioned environments. Strategies such as 3D bioprinting, mechanostimulation in bioreactors, and the use of gene-edited tenocytes are potential avenues to improve repair and restore functional performance [25,26,27].

### 3.1. Tendon Healing: General Mechanisms and Achilles-Specific Considerations

The healing of tendons, such as the AT, is a slow and gradual process. The resulting scar tissue often has inferior mechanical properties to native tendon. In contrast to bone (which heals by regeneration), the tendon cannot completely re-establish its original hierarchical microstructure, including the highly aligned collagen fibrils and highly specialised interfascicular matrix [28,29].

The tendon repair process is typically described in three overlapping, yet distinct, stages: inflammatory, proliferative, and remodelling. The inflammatory phase lasts for a few days after the injury. It is characterised by haematoma formation, the infiltration of neutrophils and macrophages, the release of pro-inflammatory cytokines (IL-1β and TNF-α) and the start of angiogenesis [30,31]. The proliferative phase, lasting the first few weeks, is characterised by the proliferation of fibroblasts and tenocytes, increased cellularity, and the production of a collagenous ECM rich in disorganised type III collagen [31,32]. The remodelling phase can last several months, while cellularity reduces, collagen fibrils realign along the load-bearing axis, type I collagen gradually replaces type III collagen, and crimp patterns reappear [10,33].

Nevertheless, the tendon rarely completely regains its previous hierarchical structure and mechanical properties. In the AT, healing is also affected by poor intrinsic vascularisation and high mechanical demands, resulting in a higher risk of rerupture and chronic tendinopathy [16,34].

### 3.2. Tissue Regeneration Strategies for the AT

The strategies involve three core principles of tissue engineering: using cells, employing scaffolds to transport these cells, and incorporating bioactive molecules to promote cell differentiation and growth. These approaches can be applied individually or in combination to address AT injuries [35].

### 3.3. Tendon Stem/Progenitor Cells (TSPCs/TDSCs)

The discovery of tendon stem/progenitor cells (TSPCs) was a pivotal breakthrough in tendon biology. TSPCs, also known as TDSCs, are a small population of cells identified in various tendons (approximately 5% of tendon cells) and characterised by their capacity for self-renewal, clonogenicity, and multipotent differentiation. TSPCs are usually located in specific ECM niches within the tendon microenvironment, where a combination of biochemical and mechanical cues regulates their behaviour and fate [36,37]. TSPCs isolated in vitro can produce tenocytes, chondrocytes, osteoblasts, and adipocytes, supporting their stem-like nature [36,38]. The inherent commitment of TSPCs to the tendon lineage is a desirable trait, as this may enable them to restore tendon tissue more faithfully than stem cells from other sources [37].

Preclinical studies have shown that TSPCs seeded on aligned nanofibre scaffolds enhance the deposition of organised collagen bundles, upregulation of tenogenic markers (such as scleraxis and tenomodulin), and biomechanical properties of repaired tendons [38,39].

However, the scarcity of TSPCs in native tendon tissue precludes large-scale harvest, and age-related decreases in the TSPC number and function may limit the therapeutic potential of TSPCs in older individuals. Therefore, methods to expand TSPCs in vitro or recruit them in situ are an active research area [37,38,40].

### 3.4. Cells in Tissue Engineering

Cells are crucial in tendon tissue engineering, providing the necessary biological activity for regeneration. Tendon-derived stem cells (TDSCs) have shown potential due to their clonogenicity, multipotency, and ability to produce type I collagen while maintaining tissue-specific differentiation, making them promising for AT repair. Research has shown that tendon tissue engineered from TDSCs can mimic the structure and mechanical characteristics of embryonic tendons. However, clinical application remains limited by the low cell density of tendon tissue and the difficulties in obtaining sufficient donor cells [41,42].

### 3.5. Tenocytes and Tenoblasts

Tenocytes are fibroblast-like cells that have undergone terminal differentiation and are the predominant cell type in adult tendon tissue. They are responsible for the continual synthesis and remodelling of type I collagen, proteoglycans, and other constituents of the ECM required for normal tendon function. The precursors to tenocytes are called tenoblasts, characterised by high proliferative potential; however, they are sparse in adult tissues [43,44].

The direct application of autologous tenocytes and tenoblasts to repair or regenerate damaged tendons appears to be a logical approach, given their native tenogenic phenotype and ability to synthesise matrix components. However, several problems have limited their use. Tenocytes can only be harvested via biopsy from a healthy tendon, resulting in donor site morbidity and a low yield of cells for research purposes. Additionally, the in vitro expansion of tenocytes has been associated with phenotypic drift, characterised by the loss of tenogenic markers and the ability to differentiate into chondrocytic and osteogenic lineages, with an increased propensity for ectopic ossification [39,45].

Therefore, while tenocytes have been helpful in experimental models, they are not an ideal or scalable source of cells for widespread clinical use [46].

### 3.6. Mesenchymal Stem/Stromal Cells (MSCs)

MSCs are multipotent, non-haematopoietic cells of mesodermal origin capable of differentiating into bone, cartilage, and adipose lineages. They have been isolated from multiple clinically relevant tissues, including bone marrow, adipose tissue, synovium, periosteum, muscle, umbilical cord, skin/peripheral blood, periodontal ligament, and tendon itself [47,48]. Owing to their accessibility and capacity for expansion, bone marrow-derived MSCs (BMSCs) and adipose-derived MSCs (ADSCs) are the most widely investigated sources for regenerative approaches. Their therapeutic potential is closely linked to their ability to undergo tenogenic differentiation when stimulated by GDF-5 (BMP-14), GDF-7 (BMP-12), TGF-β, or through biomechanical and genetic cues, which up-regulate tendon transcription factors such as scleraxis and tenomodulin and increase extracellular matrix synthesis in vitro and in vivo [48,49]. However, osteo-chondrogenic drift can occur with prolonged in vitro expansion or suboptimal induction, making careful control of culture conditions important [48].

In vivo studies support the regenerative capacity of MSC-based therapies. In a rabbit AT defect model, BMSC-seeded scaffolds generated spindle-shaped tenocytes, parallel collagen alignment, neovascularisation, and mechanical strength approaching 98% of intact tendon at 6 months, significantly outperforming acellular scaffolds [50]. Comparable improvements have been reported using ADSCs, especially when combined with GDF-5, resulting in greater cell density, more organised collagen fibres, and enhanced biomechanical and histological outcomes in rat and rabbit models [51,52].

**Figure 1:**
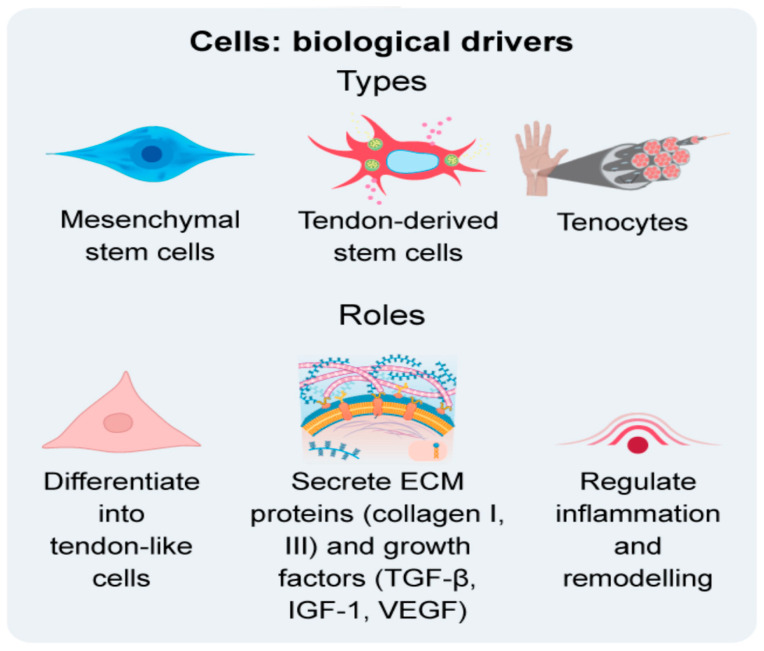
Overview of cell types and biological functions in Achilles tendon tissue engineering.

Beyond differentiation, a substantial proportion of MSC activity is mediated through paracrine mechanisms, including cytokines, growth factors, and extracellular vesicles (EVs), which modulate inflammation, promote cell proliferation and migration, and coordinate matrix remodelling [53]. EV-based strategies are emerging as a promising alternative, with BMSC-EVs improving tendon healing and collagen organisation in rat AT injuries in a dose-dependent manner [54], while ADSC-EVs enhance tenocyte proliferation, migration and extracellular matrix deposition [55]. The contribution of MSCs, tenocytes and tendon-derived stem cells to tendon regeneration is illustrated in Figure 1.

Collectively, MSCs remain central to tendon tissue engineering because they can be harvested in clinically relevant quantities, directed toward tendon lineage through biochemical and mechanical stimuli, and supplemented via EV-based approaches offering additional translational potential [48,52,53,54].

### 3.7. Scaffolds for AT Regeneration

Scaffolds are crucial in tissue engineering for ATs. They provide biomechanical support, a biomimetic ECM for cellular attachment and proliferation, and stimulation and organisation of collagen deposition and host tissue integration [34,48].

Scaffold design must be carefully considered to provide biocompatibility, biodegradability, and sufficient mechanical strength to withstand physiological loading, while promoting cellular infiltration and vascularisation and facilitating scaffold replacement by native tissue. Additional factors are also desirable, such as the incorporation of chemotactic or growth factors to attract endogenous progenitor cells, promote low immunogenicity, and complete integration into the healing tendon [33,46,55].

**Figure 2:**
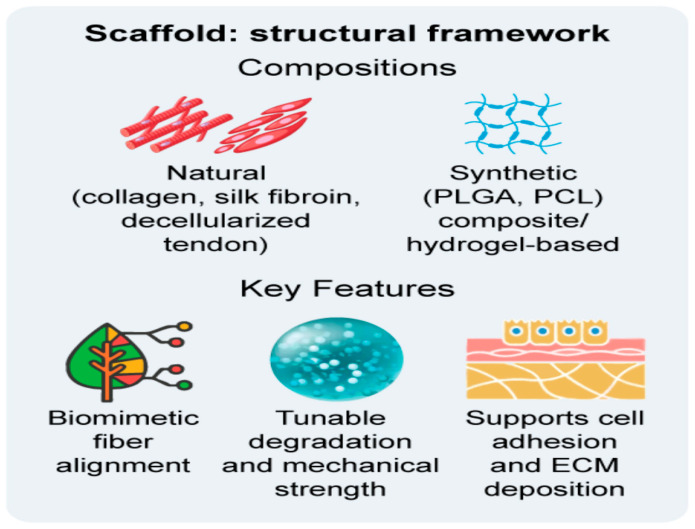
Structural features of natural, synthetic, hybrid, and decellularised scaffolds used in tendon tissue engineering.

Performance depends on not only the base material but also architecture, including fibre alignment, porosity, anisotropy, and surface chemistry. It can be further improved by mechanical preconditioning in bioreactors to promote collagen maturation and tendon-like function [33,46,56]. The main scaffold compositions and design characteristics are summarised in Figure 2.

#### 3.7.1. Natural Scaffolds

Natural scaffolds such as collagen, silk, small intestinal submucosa (SIS), gelatin, and decellularised tendons maintain the original bioactivity and cell-binding sites of the ECM. This facilitates cell adhesion and tenogenic differentiation [57,58].

Anionic collagen represents a chemically modified natural scaffold with enhanced regenerative properties. Produced through alkaline hydrolysis of native collagen, this modification converts carboxyamide groups of asparagine and glutamine residues into carboxylic groups, resulting in increased negative charge and enhanced piezoelectric properties [59,60].

Comparative studies have demonstrated that anionic collagen matrices exhibit significantly superior regeneration compared to native collagen scaffolds, with bone volume measurements showing an increase for anionic matrices versus native collagen in bone defect models [61]. The enhanced piezoelectric properties are particularly relevant for tendon tissue engineering, as piezoelectricity plays a crucial role in tendon mechanotransduction and repair signalling pathways [62]. Anionic collagen scaffolds have shown excellent biocompatibility, a low inflammatory response, and controlled biodegradation rates [59,63].

While most research has focused on bone regeneration applications, the combination of biocompatibility, enhanced piezoelectric properties, and proven superior regenerative capacity suggests a stronger potential for Achilles tendon tissue engineering.

Decellularised tendon scaffolds maintain the ECM ultrastructure and growth factors present in tendons while enabling fibroblast repopulation, with reduced immunogenicity compared to untreated grafts [64].

SIS-based matrices maintain similar bioactive molecules, including VEGF and TGF-β, recruit marrow-derived progenitors, and develop into collagen-rich, tendon-like tissue in vivo within 3 months [58].

In cadaveric studies, acellular human dermal allografts have also been found to augment stiffness and strength when used in AT repair and have shown promise in a preliminary clinical trial [65].

The use of natural scaffolds is associated with several advantages, such as including strong biological signalling and the presence of native ECM components that can be augmented to improve cell adhesion, proliferation, and tenogenic differentiation. However, natural scaffolds are often associated with batch-to-batch variability, may degrade too rapidly to provide long-term mechanical support, and can be associated with residual immunogenic material if the decellularisation process was inadequate, which can lead to an inflammatory response that impairs healing [57,64].

#### 3.7.2. Synthetic Scaffolds

Synthetic scaffolds have been extensively investigated for applications in AT tissue engineering. These biomaterials can be tailored to possess appropriate mechanical properties, defined compositions, reproducibility, and mass-production potential [66].

Electrospun polymeric scaffolds, and particularly biodegradable polyesters, poly(l-lactic acid) [PLLA], poly(lactic-co-glycolic acid) [PLGA], and polycaprolactone [PCL], can mimic the fibrous architecture of tendon ECM, which is beneficial to cell alignment and tenogenic differentiation [66,67].

Synthetic scaffolds enable precise control over material characteristics such as fibre diameter, pore size, and degradation rate and thus the fine-tuning of scaffold performance to match tendon mechanical requirements. For example, PLLA/PLGA electrospun fibres were shown to provide appropriate tensile properties while supporting MSC adhesion and proliferation [67,68].

Although synthetic scaffolds have many desirable properties for tendon tissue engineering, their use presents limitations. For example, the degradation by-products, particularly the acidic products of polyesters, can elicit an inflammatory response and impair healing [69,70]. Additionally, the lack of biological recognition sites reduces their capacity to promote cell adhesion and integration relative to ECM-derived natural scaffolds [70,71].

#### 3.7.3. Composite and Hybrid Scaffolds

Composite and hybrid scaffolds have been developed to overcome the limitations associated with the use of purely natural or synthetic biomaterials. Composite scaffolds combine the mechanical and structural properties of synthetic polymers with the biological recognition and cellular interaction of natural molecules [67]. Hybrid scaffolds, such as PCL with collagen or silk fibroin, have been shown to improve cell adhesion, proliferation, and ECM production in comparison to PCL-only constructs while retaining the required mechanical integrity for tendon regeneration [72]. Chitosan–PCL composites have also demonstrated improved biocompatibility and tenogenic differentiation capacity, attributed to the bioactive chitosan component. These systems are advantageous in that they enable fine-tuning of the degradation rates and fibre architecture to align with cues present in the native tendon ultrastructure [73].

Recent studies have emphasised the design of more complex scaffold architectures, such as multilayered or gradient scaffolds, which are better suited to replicate the tendon-bone interface in AT repair. This concept enables more accurate representation of the anisotropic mechanical environment in the native tendon, improving insertion site integration [74].

However, challenges associated with composite scaffolds include increased manufacturing complexity, potential batch-to-batch variability when natural polymers are incorporated, and issues with scaling up production processes. Nevertheless, the capacity of hybrid scaffolds to harness the benefits of natural and synthetic materials renders this approach among the most promising strategies for scaffold-based tendon regeneration [48,75].

#### 3.7.4. Hydrogel Scaffolds

Hydrogels can provide hydrated ECM-mimicking environments conducive to the encapsulation and delivery of cells and biofactors and have also emerged as an attractive approach to AT repair [33,48]. Injectable materials, such as gelatin methacrylate (GelMA), PEG, fibrin, and hyaluronic acid derivatives, can be used to fill irregular defects in a minimally invasive manner [34,46]. They have also been used to deliver growth factors (TGF-β, GDF-5/7, PDGF, and IGF-1) and transplanted cells to improve tenogenic differentiation and angiogenesis, as well as matrix deposition [33,48]. Alone, hydrogels have limited tensile strength for AT loading and can result in swelling-induced stress [33,48]. This has resulted in a trend towards the use of hydrogels in combination with fibrous scaffolds, nanofibres, or composite designs to improve mechanical integrity while maintaining biological activity [34,46]. A comparative overview of scaffold categories is presented in Table 1. Additional comparative data on scaffold categories can be found in the Supplementary Materials (Table S2).

### 3.8. Bioactive Molecules and Biologics in Tendon Repair

Bioactive molecules, including growth factors, cytokines, chemokines, nucleic acids, and hormones, modulate cell growth, differentiation, and ECM synthesis during tendon healing and regeneration [34,46,77]. Recombinant growth factors are desirable because they can be produced with high purity, delivered in defined doses, and incorporated into engineered systems [46,48]. In tendon tissue engineering, growth factors are delivered directly (in solution or by injection), in combination with platelet concentrates, or immobilised/encapsulated within scaffolds to control release kinetics and local bioavailability [46,48,71].

Key molecules implicated in tenogenesis and tendon repair include TGF-β isoforms, PDGF-BB, IGF-1, FGF-2, VEGF, and BMP-12/13/14 (also known as GDF-7, 6, and 5) [46,48,78]. TGF-β signalling promotes tenogenic gene expression and collagen synthesis; however, prolonged activity can lead to fibrosis and adhesions, highlighting the importance of controlled delivery [79,80]. PDGF-BB drives the chemotaxis and proliferation of tendon fibroblasts and MSCs and provides angiogenic support during early healing [78,81]. IGF-1 stimulates tenocyte proliferation and collagen production and has been studied extensively as a tendon-healing adjunct [82,83]. FGF-2 supports cell proliferation, neovascularisation, and ECM remodelling, although its strong angiogenic effects must be tightly regulated to prevent disorganised scar tissue [84]. VEGF is critical to the revascularisation of the hypovascular AT; however, unregulated exposure may impair matrix quality. Thus, gradient or staged-delivery strategies have been developed to limit its action to early healing stages [76,85].

Autologous platelet-rich plasma (PRP) provides a combination of endogenous growth factors (PDGF, TGF-β, VEGF). However, high-quality randomised trials in Achilles tendinopathy and acute rupture have shown limited or no functional benefit, probably due to variability in formulations and protocols [86,87]. Recent consensus highlights the need for standardised PRP characterisation to achieve more reproducible and clinically comparable results [88].

In addition to proteins, small molecules and bioactive peptides are increasingly being used as signalling stimulators because of their stability, lower cost, and scalability. Because tendon repair is inherently mechanobiological, combining scaffold-based bioactive factor delivery with controlled mechanical loading is essential to direct collagen alignment and restore functional biomechanics [34,46,48].

### 3.9. Load in AT Tissue Engineering

Mechanical loading is a well-documented aspect of tendon biology with important implications for AT tissue engineering strategies. Tendons are known to be mechanosensitive, with tenocytes and TSPCs modulating gene expression, ECM production, and collagen fibrillogenesis and alignment in response to mechanical loading [2,89,90].

The AT experiences large mechanical loads, with peak forces reaching 12.5 times the body weight during running; thus, recapitulating physiological loading is a critical component of tissue-engineered tendon constructs [91]. The primary physiological loading stimulus for the AT is tensile loading, a key regulator of cellular differentiation and matrix organisation. The magnitude of tensile strain can direct transcription factor activation, including the activation of scleraxis, a transcription factor involved in early collagen fibrillogenesis, and mohawk, a transcription factor known to regulate fibril diameter during fibril growth and maturation [92].

In vitro studies have demonstrated that cyclic tensile stimulation enhances type I collagen deposition and fibril alignment along the loading axis compared to static culture, promoting superior mechanical strength [93].

However, a lack of tensile load produces scar-like tissue characterised by randomly oriented collagen fibrils. Thus, the control of mechanostimulation is a key component of regenerative strategies [94].

In an experimental model, the ablation of physiological compression in tendons resulted in reduced proteoglycan content and decreased compressive stiffness, indicating an important role for compressive cues in maintaining the tendon-bone interface [95,96].

Shear forces represent another class of mechanical stimuli relevant to tendon biology. Fascicular sliding in the AT is well-documented, and interfascicular shear enables the redistribution of stress during loading, contributing to the tendon’s resistance to rupture [97].

Ageing tendons, which exhibit decreased fascicular sliding, are associated with increased stiffness and susceptibility to rupture, suggesting a role for shear adaptation in these processes [98].

Lubricin, a proteoglycan known to be upregulated at interfaces experiencing shear, has been shown to promote fascicular gliding and is a demonstrated modulator of tendon mechanobiology [99].

Constructs enabling directed shear adaptation, by scaffold architecture or bioreactor conditioning, may improve the long-term durability of engineered AT [90,100].

The mechanotransduction pathways in response to loading identified at the molecular level include integrin signalling, stretch-activated ion channels, and their downstream pathways (MAPK/ERK and Wnt/β-catenin), which control proliferation, differentiation, and ECM remodelling [89,101].

The effects of mechanical loading can be positive and negative. Specifically, underloading or overloading is not beneficial and results in degenerative changes. An insufficient amount of mechanical stimulation leads to underdeveloped tissue. However, overloading increases catabolic activity, for instance, the increased production of MMPs and the pathological accumulation of collagen III. Therefore, a specific loading regimen is required for AT tissue engineering to encourage regenerative effects [102].

**Figure 3:**
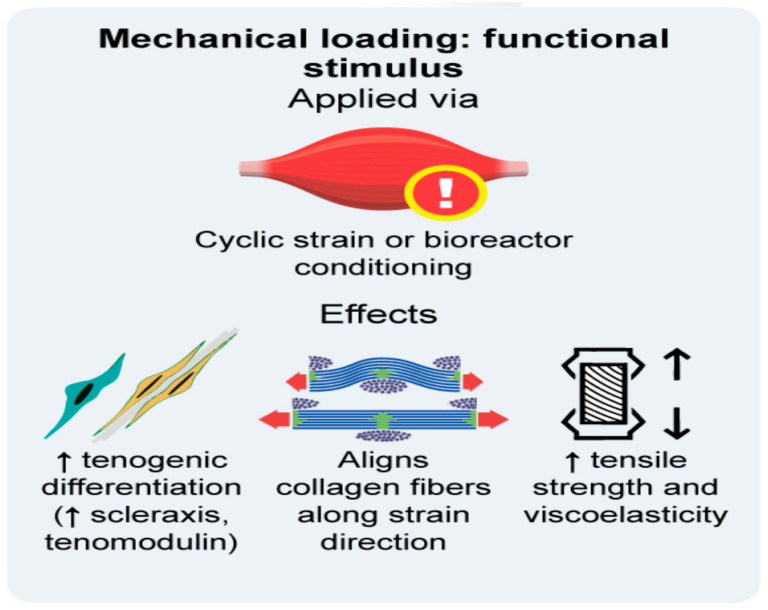
Mechanical loading as a functional stimulus and its cellular and extracellular effects.

Consequently, mechanical loading through tension, compression, and shear stress is not only crucial for biomechanical reasons; it also contributes to the phenotype of the tendon construct. Applying physiological and stage-specific loading conditions in the bioreactor or scaffold is therefore essential to engineer a tendon with a hierarchical organisation, zonal distribution, and the native mechanical properties of the AT [17,90,103]. The effects of bioreactor conditioning and mechanical stimulation are shown in Figure 3.

### 3.10. Advanced Fabrication Techniques

Although the choice of scaffold materials is important, the method used to fabricate these biomaterials into functional constructs is equally critical to the final scaffold’s architecture, mechanical properties, and biological performance [48].

One of the most significant innovations in tendon tissue engineering is the ability to customise the architecture and distribution of cell layers with controlled fibre alignment using 3D bioprinting technology. Bioprinting of tendon tissues is normally performed using extrusion-based techniques [27,104].

Extrusion-based bioprinting, the most widely adopted technique for tendon applications, uses shear-thinning bioinks comprising cell-embedded collagen, GelMA, and decellularised tendon ECM to construct and place design fibres that mimic the anisotropic structural alignment of the native tendon [105].

Studies have shown that bioprinted scaffolds seeded with MSCs or TSPCs exhibit better collagen deposition, mechanical properties, and tenogenic marker (scleraxis and tenomodulin) activation than scaffolds produced using traditional fabrication methods [106].

Three-dimensional bioprinting can design constructs based solely on medical imaging data, enabling easy personalisation of the AT and making this technology extremely useful and versatile [48,104].

### 3.11. Clinical Applications and Outcomes

Although promising results have been achieved in animal models and considerable advances have been made in preclinical research, the clinical translation of tissue engineering approaches for AT repair remains at an early developmental stage [107].

The translation of cell-based and scaffold-based therapies is further complicated by stringent regulatory requirements, as these products are classified as advanced therapy medicinal products (ATMPs) by agencies such as the FDA, EMA, and MHRA, requiring rigorous safety and efficacy data [108,109].

Among other approaches, mesenchymal (stem) cell therapy has garnered the most attention in the treatment of Achilles tendinopathy, as MSCs can modulate inflammation and matrix remodelling and differentiate into tenogenic progeny [110].

A Phase IIa clinical trial by Goldberg et al. (2024) demonstrated that, after autologous bone marrow-derived MSCs were injected into the ATs of 10 patients with chronic mid-portion tendinopathy, no serious side effects were reported. Considerable improvements in VISA-A scores at 6 and 12 months were observed in pain and other aspects of function, indicating that the treatment is safe [111].

However, a systematic review by van den Boom et al. (2020) concluded that, while Level 3 evidence supports the efficacy of stem cell therapy for tendon disorders, the findings are at considerable risk of bias due to small sample sizes, a lack of control groups, and heterogeneity in cell preparation and delivery methods [112]. Significant challenges include a lack of standardised MSC isolation and culture protocols, variable cell doses, inconsistent injection techniques, and the absence of large-scale randomised controlled trials [113].

Several acellular scaffold products have received regulatory clearance for soft tissue augmentation in tendon repair, including GraftJacket (acellular human dermal matrix), TissueMend (fetal bovine dermis), and REGENETEN (bioinductive bovine collagen scaffold) [114].

Cadaveric biomechanical studies, such as that by Barber et al. (2008), have demonstrated that the augmentation of AT repairs with GraftJacket significantly increases repair strength and stiffness compared to repair alone [65].

Recent case series by Ling et al. (2024) and Genuth et al. (2025) reported that bioinductive collagen scaffold augmentation for AT ruptures and insertional tendinitis appears safe and feasible, with good functional outcomes and no increase in complication rates [115,116].

However, the absence of randomised controlled trials focusing on scaffold-augmented repair in comparison to traditional repair methods precludes truly conclusive, clinically impactful findings, warranting more targeted research [48].

The direct application of certain recombinant growth factors, such as PDGF-BB, IGF-1, and the BMPs, has been considered to enhance tendon healing by stimulating the proliferation of tendon cells, as well as the matrix and collagen structures associated with tendons [76].

Although growth factor injections have shown promising results in preclinical studies, clinical translation has been limited by challenges such as the short half-life of recombinant proteins, difficulty in achieving sustained local delivery, and a lack of large-scale clinical trials specifically in the context of AT repair [76].

Future strategies may include controlled-release delivery systems, such as growth factor-loaded scaffolds, which could maintain therapeutic concentrations over extended periods and potentially enhance clinical efficacy [76,117]. Key preclinical and clinical studies are summarized in Table 2. Further detailed study characteristics are available in the Supplementary Materials (Table S1).

### 3.12. Current Limitations and Challenges

A major obstacle to broader clinical adoption remains the striking lack of consistency across published studies. Patient selection criteria, treatment protocols, and follow-up periods vary considerably between studies, making cross-study comparisons and robust meta-analyses exceptionally challenging [118,119]. This problem is compounded by the absence of standardised outcome measures; some studies rely on patient-reported measures, such as VISA-A or AOFAS scores, while others emphasise imaging modalities such as ultrasound or MRI, and still others focus on biomechanical testing. Such heterogeneity hinders efforts to synthesise evidence and develop clear clinical guidelines [120].

Another limitation concerns the temporal scope of available data. Most clinical trials report outcomes over relatively short to medium time frames, typically between 6 and 24 months, with surprisingly minimal information on long-term durability or rerupture rates. Without extended follow-up data, it is challenging to assess whether early improvements are sustained or whether complications emerge over time [112,113].

Finally, economic and regulatory factors cannot be overlooked. Cell-based and scaffold-augmented therapies tend to be expensive, and insurance coverage remains limited. Navigating the regulatory landscape for ATMPs adds further complexity. These barriers restrict widespread clinical adoption and limit patient access to potentially beneficial treatments [121,122].

Standardization of cell isolation, expansion, and delivery protocols is crucial to reduce variability across studies and achieve reproducible clinical outcomes. The establishment of multicenter registries and adherence to international reporting standards, such as the ICON Tendinopathy Consensus, may facilitate harmonization and meta-analytic comparisons [120].

Therefore, although tissue engineering strategies for AT repair have demonstrated safety and feasibility in early-phase clinical studies, definitive evidence of clinical superiority over conventional surgical or conservative treatments remains elusive [48,71].

**Figure 4:**
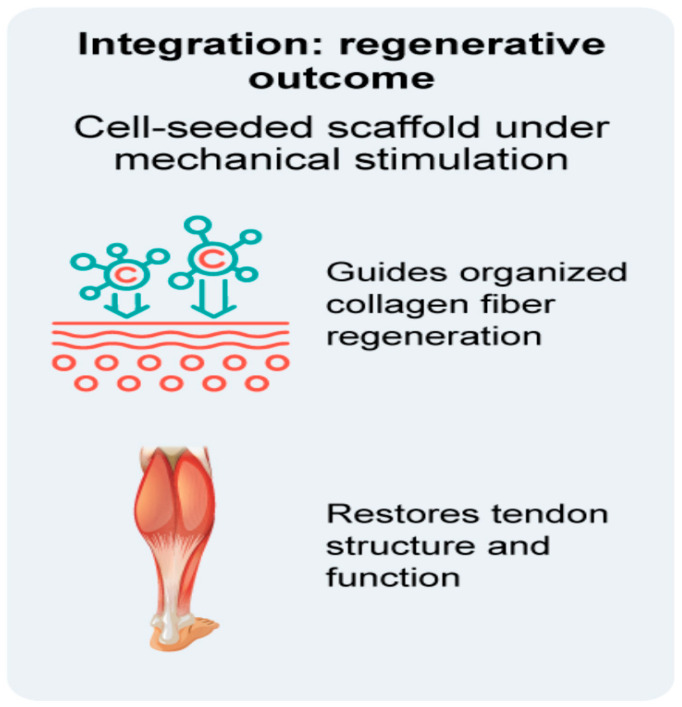
Integrated tissue engineering approach for restoring Achilles tendon structure and function.

A schematic representation of the integrated regenerative approach is provided in Figure 4.

### 3.13. Future Directions in Tissue Engineering

Despite the challenges outlined previously, tendon tissue engineering is at an inspiring time, with several novel technologies and strategies emerging with the potential to overcome many of these limitations [71,123].

In the future, AT tissue engineering is likely to be shaped by several key transformative technologies and approaches, including biofabrication, molecular therapeutics, and precision medicine [104,119].

Overcoming the translational problems outlined previously and approaching clinically relevant outcomes will require the next wave of tissue engineering strategies to incorporate advances from various disciplines, including materials science, biofabrication, genomics, and precision medicine [48,119].

One of the most promising areas of development is the design of patient-specific 3D bioprinted tendon grafts, which can be customised based on patient anatomy and injury parameters using medical imaging data (CT, MRI) [104,124].

Additionally, the next generation of biomaterials is likely to include “smart” or stimuli-responsive materials that can dynamically respond to the healing environment, providing control over growth factor delivery. An example is scaffolds with mechanically responsive nanoparticles that release therapeutic molecules in response to tensile loading, synchronising drug delivery with the timing of rehabilitation protocols [110,125,126].

Such controlled-release systems can overcome a significant limitation of direct growth factor injection, with the short half-life of recombinant proteins, by providing sustained release of therapeutics over extended periods [125].

## 4. Conclusions

This review consolidates the current advancements in AT tissue engineering, highlighting progress in cellular therapy, scaffold biomaterials, and mechanobiology, all aimed at achieving functional regeneration. Our primary findings reveal that the field has evolved significantly from basic biological constructs to intricate multicomponent systems. Preclinical research consistently shows the promise of this approach, with notable results indicating that MSC-seeded scaffolds can restore biomechanical strength close to native levels and that chemically modified scaffolds can significantly surpass native collagen in facilitating tissue formation. Initial clinical trials have confirmed the safety of cell-based therapies and the practicality of scaffold enhancement, with reports of notable functional improvements in patients suffering from chronic tendinopathy. Despite these advancements, a considerable gap in translation remains. The clinical outlook is cautiously optimistic, hindered by the absence of large-scale randomized controlled trials, variability in treatment protocols, and a lack of standardized outcome measures. Therefore, overcoming these obstacles is crucial for future clinical success. The future clinical perspective involves a comprehensive strategy: establishing a global consensus on manufacturing protocols and clinical endpoints, designing more intelligent and efficient clinical trials, and creating patient-specific solutions through technologies like 3D bioprinting. Ultimately, converting the remarkable laboratory successes into sustainable, accessible clinical therapies will require ongoing interdisciplinary collaboration among clinicians, engineers, and biologists to navigate the complex path from scientific discovery to a new standard of care for Achilles tendon injuries.

## Figures and Tables

**Table 1:** Comparative overview of natural, synthetic, hybrid, hydrogel, and decellularised anionic collagen scaffolds employed in Achilles tendon tissue engineering. The table summarises principal materials, key advantages, limitations, and representative literature supporting each category.

Scaffold Type	Common Materials	Advantages	Limitations	Representative Studies
Natural scaffolds	Collagen, silk fibroin, gelatin, SIS, decellularised tendon	Excellent biocompatibility and cell adhesion; retain ECM bioactivity; support tenogenic differentiation	Variable degradation rates; potential immunogenicity; limited mechanical strength	Rieu et al., 2015; Badylak et al., 1998; Farnebo et al., 2014 [57,58,59]
Synthetic scaffolds	PLLA, PLGA, PCL, PHBHHx	Tunable mechanical properties; reproducible; scalable manufacturing	Lack of bioactive motifs; acidic degradation by- products may trigger inflammation	Reverchon et al., 2012; Zhang et al., 2023; Heidari et al., 2023 [61,62,64]
Hybrid/composite scaffolds	PCL–collagen, PCL–silk, chitosan–PCL, multilayered gradient designs	Combine biological recognition with mechanical strength; adjustable degradation and architecture	Complex fabrication; potential batch variation	Leung et al., 2013; Emonts et al., 2024; Song et al., 2025 [67,68,69]
Hydrogel scaffolds	GelMA, PEG, fibrin, hyaluronic acid derivatives	Injectable; mimic hydrated ECM; allow growth-factor or cell encapsulation	Low tensile strength; swelling stress; often require fibre reinforcement	Zhu et al., 2022; Lin et al., 2023 [34,76]
Decellularised Anionic Collagen (from AT)	Modified Achilles tendon collagen with increased surface charge	Enhanced cell adhesion and proliferation; preserved ultrastructure; reduced immunogenicity	Limited clinical data; scalability challenges	Rieu et al., 2015; Farnebo et al., 2014 [57,59]

**Table 2:** Summary of key preclinical and clinical studies on Achilles tendon (calcaneal tendon) tissue engineering using the PICOS framework. The table highlights model populations, interventions, comparators, primary outcomes, and concise summaries of findings relevant to regenerative performance.

Study	Population	Intervention	Comparison	Outcomes	Summary
Chailakhya n et al., 2021 [50]	Rabbit Achilles tendon defect	Bone marrow– derived MSC- seeded scaffold	Cell-free scaffold	Tensile strength, collagen alignment	Achieved ~98% of intact tendon strength after 6 months; superior biomechanica l recovery.
de Aro et al., 2018 [51]	Rat Achilles Tendon defect	Adipose- derived stem cells (ADSCs) + GDF-5	ADSCs alone	Histological organisation, biomechanica l recovery	Increased collagen alignment, cellularity, and mechanical strength in ADSC + GDF- 5 group.
Goldberg et al., 2024 [111]	Human chronic mid- portion tendinopathy	Autologous bone marrow– derived MSC injection	None (open- label trial)	VISA-A score improvement, safety	Significant pain and function improvement at 6–12 months; no major adverse events
Ling et al., 2024 [115]	Patients with acute AT rupture	Bioinductive collagen scaffold augmentation	Conventional repair	Functional outcome, complication rate	Good postoperative recovery and comparable complication rate to control; promising feasibility
Xie et al., 2019 [52]	Rabbit AT defect	Decellularised tendon matrix scaffold + BMSC sheet	Decellularised scaffold only	Collagen organisation, mechanical properties	Improved fibre alignment, renovated morphology, and mechanical stiffness

## Data Availability

No new data were created or analyzed in this study.

## Supplementary Materials

The following supporting information can be downloaded at: https://www.mdpi.com/article/10.3390/bioengineering12121346/s1, Table S1: Summary of Key Studies on Achilles Tendon Tissue Engineering (PICOS Framework); Table S2: Comparison of Biomaterial Scaffold Types for Achilles Tendon Tissue Engineering.
