# Supplementary material for: Advances in Achilles Tendon Tissue Engineering: Integrating Cells, Scaffolds, and Mechanical Loading for Functional Regeneration
Source: Bioengineering (Basel). 2025 Dec 10;12(12):1346. doi: 10.3390/bioengineering12121346 (PMC12730031; doi:10.3390/bioengineering12121346)
Supplement: Supplementary file 1 [file bioengineering-12-01346-s001.zip › bioengineering-3965423-supplementary.pdf]

Tables and Legends for Achilles Tendon Tissue Engineering Manuscript

Table S1. Summary of Key Studies on Achilles Tendon Tissue Engineering (PICOS Framework)

Table S1. Summary of key preclinical and clinical studies on Achilles tendon (calcaneal tendon) tissue engineering using the PICOS framework. The table highlights model populations, interventions, comparators, primary outcomes, and concise summaries of findings relevant to regenerative performance.

Study	Population	Intervention	Comparison	Outcomes	Summary
Chailakhyan et al., 2021	Rabbit Achilles tendon defect	Bone marrow–derived MSC-seeded scaffold	Cell-free scaffold	Tensile strength, collagen alignment	Achieved ~98% of intact tendon strength after 6 months; superior biomechanical recovery.
de Aro et al., 2018	Rat Achilles tendon defect	Adipose-derived stem cells (ADSCs) + GDF-5	ADSCs alone	Histological organisation, biomechanical recovery	Increased collagen alignment, cellularity, and mechanical strength in ADSC + GDF-5 group.
Goldberg et al., 2024	Human chronic mid-portion tendinopathy	Autologous bone marrow–derived MSC injection	None (open-label trial)	VISA-A score improvement, safety	Significant pain and function improvement at 6–12 months; no major adverse events.
Ling et al., 2024	Patients with acute AT rupture	Bioinductive collagen scaffold augmentation	Conventional repair	Functional outcome, complication rate	Good postoperative recovery and comparable complication rate to control; promising feasibility.
Xie et al.,	Rabbit AT	Decellularise	Decellularise	Collagen	Improved

2019	defect	d tendon matrix scaffold + BMSC sheet	d scaffold only	organisation, mechanical properties	fibre alignment, tenocyte morphology, and mechanical stiffness.

References

- Chailakhyan R K et al. Int Orthop. 2021; 45:3263–3276.
- de Aro A A et al. PLoS ONE. 2018; 13:e0202514.
- Goldberg A J et al. Front Bioeng Biotechnol. 2024; 12:1357871.
- Ling J et al. Front Bioeng Biotechnol. 2024; 12:1580490.
- Xie S et al. J Orthop Res. 2019; 37:887–897.

Table S2. Comparison of Biomaterial Scaffold Types for Achilles Tendon Tissue Engineering

Table S2. Comparative overview of natural, synthetic, hybrid, hydrogel, and decellularised anionic collagen scaffolds employed in Achilles tendon tissue engineering. The table summarises principal materials, key advantages, limitations, and representative literature supporting each category.

Scaffold Type	Common Materials	Advantages	Limitations	Representative Studies
Natural Scaffolds	Collagen, silk fibroin, gelatin, SIS, decellularised tendon	Excellent biocompatibility and cell adhesion; retain ECM bioactivity; support tenogenic differentiation	Variable degradation rates; potential immunogenicity; limited mechanical strength	Rieu et al., 2015; Badylak et al., 1998; Farnebo et al., 2014
Synthetic Scaffolds	PLLA, PLGA, PCL, PHBHHx	Tunable mechanical properties; reproducible; scalable manufacturing	Lack of bioactive motifs; acidic degradation by-products may trigger inflammation	Reverchon et al., 2012; Zhang et al., 2023; Heidari et al., 2023
Hybrid / Composite Scaffolds	PCL-collagen, PCL-silk, chitosan-PCL, multilayered gradient designs	Combine biological recognition with mechanical strength; adjustable degradation and architecture	Complex fabrication; potential batch variation	Leung et al., 2013; Emonts et al., 2024; Song et al., 2025
Hydrogel Scaffolds	GelMA, PEG, fibrin, hyaluronic acid derivatives	Injectable; mimic hydrated ECM; allow growth-factor or cell encapsulation	Low tensile strength; swelling stress; often require fibre reinforcement	Zhu et al., 2022; Lin et al., 2023
Decellularised Anionic Collagen (from AT)	Modified Achilles tendon collagen with increased surface charge	Enhanced cell adhesion and proliferation; preserved ultrastructure; reduced immunogenicity	Limited clinical data; scalability challenges	Rieu et al., 2015; Farnebo et al., 2014

References

- Rieu C et al. Curr Org Chem. 2015; 19:1552–1574.
- Badylak S F et al. Tissue Eng. 1998; 4(4):361–373.

- Farnebo S et al. Plast Reconstr Surg. 2014; 133(1):79–89.
- Reverchon E et al. Muscles Ligaments Tendons J. 2012; 2:181–186.
- Zhang Y et al. Polymers. 2023; 15(6):1566.
- Heidari B S et al. Bioact Mater. 2023; 19:179–197.
- Leung M et al. J Mater Chem B. 2013; 1(47):6516–6524.
- Emonts C et al. Polymers. 2024; 16(16):2349.
- Song T et al. Nano-Micro Lett. 2025; 17:Article ID xxx.
- Lin M et al. Front Bioeng Biotechnol. 2023; 11:1250533.
